# *Kratosvirus quantuckense*: the history and novelty of an algal bloom disrupting virus and a model for giant virus research

**DOI:** 10.3389/fmicb.2023.1284617

**Published:** 2023-11-30

**Authors:** Alexander R. Truchon, Emily E. Chase, Eric R. Gann, Mohammad Moniruzzaman, Brooke A. Creasey, Frank O. Aylward, Chuan Xiao, Christopher J. Gobler, Steven W. Wilhelm

**Affiliations:** ^1^Department of Microbiology, University of Tennessee, Knoxville, Knoxville, TN, United States; ^2^The Henry M. Jackson Foundation for the Advancement of Military Medicine, Inc., Bethesda, MD, United States; ^3^Department of Surgery, Uniformed Services University of the Health Sciences, Bethesda, MD, United States; ^4^Surgical Critical Care Initiative (SC2i), Department of Surgery, Uniformed Services University of the Health Sciences, Bethesda, MD, United States; ^5^Department of Marine Biology and Ecology, University of Miami, Miami, FL, United States; ^6^Department of Biological Sciences, Virginia Tech, Blacksburg, VA, United States; ^7^Department of Chemistry and Biochemistry, The University of Texas at El Paso, El Paso, TX, United States; ^8^School of Marine and Atmospheric Sciences, Stony Brook, NY, United States

**Keywords:** harmful algal blooms, *Nucleocytoviricota*, strain heterogeneity, model system, marine microbiology, viral ecology, *Aureococcus anophagefferens*, brown tide

## Abstract

Since the discovery of the first “giant virus,” particular attention has been paid toward isolating and culturing these large DNA viruses through *Acanthamoeba* spp. bait systems. While this method has allowed for the discovery of plenty novel viruses in the *Nucleocytoviricota*, environmental -omics-based analyses have shown that there is a wealth of diversity among this phylum, particularly in marine datasets. The prevalence of these viruses in metatranscriptomes points toward their ecological importance in nutrient turnover in our oceans and as such, in depth study into non-amoebal *Nucleocytoviricota* should be considered a focal point in viral ecology. In this review, we report on *Kratosvirus quantuckense* (née Aureococcus anophagefferens Virus), an algae-infecting virus of the *Imitervirales*. Current systems for study in the *Nucleocytoviricota* differ significantly from this virus and its relatives, and a litany of trade-offs within physiology, coding potential, and ecology compared to these other viruses reveal the importance of *K. quantuckense*. Herein, we review the research that has been performed on this virus as well as its potential as a model system for algal-virus interactions.

## Introduction

Eukaryotic phytoplankton are important contributors to nutrient cycling in marine systems (Olsen, 1989; Bratbak et al., 1993; Gobler et al., 1997). In global oceans, a diverse consortium of eukaryotic algae drives biogeochemical processes through photosynthetic sequestration of CO_2_ (Worden et al., 2012; Pierella Karlusich et al., 2020). In some contexts, these single-celled organisms can grow to densities an order of magnitude or more beyond baseline biomass levels and act as a carbon sink as well as a reservoir or source of other organic nutrients (Proctor and Fuhrman, 1991; Fagerbakke et al., 1996). These dense populations (colloquially known as “blooms”) eventually collapse, leading to the reintroduction of dissolved and particulate organic matter into the water column (Gobler et al., 1997; Fuhrman, 1999). Of specific interest in aquatic ecosystems are phytoplankton proliferations that are detrimental to ecosystems colloquially known as harmful algal blooms (HABs). This can occur through processes including consumption of nutrients, production of secondary metabolites which can act as toxins that impact other organisms, attenuation of light, or a combination of these effects (Landsberg, 2002). Bloom collapse and subsequent consumption of released organic bloom matter by heterotrophic bacteria can lead to the generation of hypoxic zones that negatively impact macrofauna (Watson et al., 2016; Anderson et al., 2021).

Understanding the drivers of algal bloom dynamics is a path toward mitigating their damage. An emerging model organism for such studies is *Aureococcus anophagefferens*, a unicellular pelagophyte that is a causative agent of brown tides endemic to bays in the eastern United States, China, and South Africa (Sieburth et al., 1988; Probyn et al., 2001; Zhang et al., 2012). Brown tides typically form in the late spring and reach peak density in early summer, before crashing mid-summer after which the microbial population of the water column is overtaken by heterotrophic bacteria (Gobler et al., 2011). *A. anophagefferens* is the host to a large DNA virus originally named Aureococcus anophagefferens Virus (AaV) (Rowe et al., 2008), and now classified into the species *Kratosvirus quantuckense* (Aylward et al., 2023). Evidence suggests that this virus is at least partially responsible for the collapse of annual HABs formed by *A. anophagefferens* (Gastrich et al., 2004; Gann et al., 2021) as has been described by viruses of other HAB and non-HAB algae (Schroeder et al., 2002; Brussaard et al., 2004; Maruyama and Ueki, 2016).

Since the isolation of *K. quantuckense*, a combination of *in vitro*, *in situ*, and *in silico* analyses have been performed to elucidate the role it plays in modulating HAB dynamics. Significant progress has been made to improve assays used to characterize infection of *A. anophagefferens* and virus propagation (Table 1). In this review, we seek to update known observations of this novel host-virus system and place their importance in context to further our understanding of HAB-virus interactions. We propose the *A. anophagefferens*—*K. quantuckense* culture-based approach as a valid model system for further research into HAB dynamics as well as the ecology of underrepresented clades of the *Nucleocytoviricota*.

**TABLE 1:** A curated table of methods for studying the host-virus system of *K. quantuckense* and *A. anophagefferens*.

Protocol name	Relevant DOI or link	Summary
**Algae related**		
Cell counting by CytoFLEX	doi.org/10.17504/protocols.io.q26g7yby9gwz/v1	High throughput method to determine *A. anophagefferens* cells abundance and other descriptive measurements such as relative size (Violet Side Scatter channel approach).
Cell and population characterization by FlowCam	doi.org/10.17504/protocols.io.14egn2336g5d/v1	A method for characterizing cells physically and to a high number of culture/sample images
Photosynthetic efficiency and quantum yield of culture	doi.org/10.17504/protocols.io.yxmvm2785g3p/v1	A sensitive method for evaluating photosynthesis of *A. anophagefferens* cultures under different treatments.
Culture maintenance media	doi.org/10.17504/protocols.io.g29byh6	A tried and true media used for the culturing of *A. anophagefferens* for both maintenance and experimental setup
Electroporation for plasmid insertion	doi.org/10.17504/protocols.io.g2vbye6	Successfully insertion of a plasmid into *A. anophagefferens* by application of electrical pulse (i.e., physical transfection)
**Virus related**		
Viral particle counting by CytoFLEX	doi.org/10.17504/protocols.io.dm6gpj331gzp/v1	A high throughput method of determining viral particle counts in a population or lysate by SYBR Gold staining.
Infectivity of viral particles by plaque assay	doi.org/10.17504/protocols.io.g2nbyde	A simple plate-based method that permits estimates of *K. quantuckense* viral particle infectivity.
Infectivity of viral particles by most probably number	doi.org/10.17504/protocols.io.gunbwve	A SpectraMax based method that permits estimates of *K. quantuckense* viral particle infectivity.
Major capsid protein copy count determination	doi.org/10.1371/journal.pone.0226758	A qPCR-based approach to confirming *K. quantuckense* MCP in culture based or *in situ* samples.
High molecular weight viral DNA extraction	doi.org/10.17504/protocols.io.n2bvjx7owlk5/v1	Extraction of *K. quantuckense* nucleic acid at high throughput sequencing quality

## An ecologically relevant alga

**FIGURE 1:**
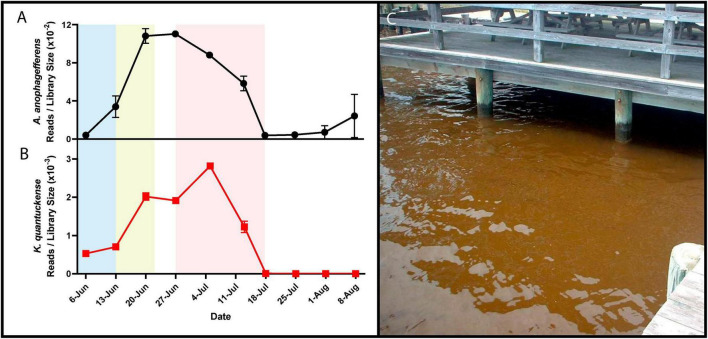
RNA reads mapped to *A. anophagefferens* **(A)** and *K. quantuckense* **(B)** coding regions normalized by library size throughout the stages of a brown tide bloom. Shaded regions depict the bloom’s initiation (blue), exponential growth (yellow), and collapse and termination (red). A dense brown tide bloom off the coast of Long Island, NY **(C)**.

For our purposes, an algal bloom can be defined as a rapid proliferation of one or more dominant species of phytoplankton in excess of the mortality normally caused by natural processes (both biotically and abiotically) (Buskey et al., 1997; Schleyer and Vardi, 2020). Blooms generally follow an “initiation, exponential growth, and termination” pattern (Schleyer and Vardi, 2020), as well as occasional maintenance stages of steady photosynthetic activity before eventual collapse (Weisberg et al., 2016; Tang et al., 2018; Zepernick et al., 2021; Figure 1A). *A. anophagefferens* blooms have been documented annually in the northeastern United States since the 1980s, particularly around Long Island, NY, and is frequently detected in other coastal waters of the country (Sieburth et al., 1988; Gobler and Sunda, 2012). It has been proposed that a 1985 bloom in Barnegat Bay (New Jersey) was previously misidentified as *Nannochloris atomus* and was *A. anophagefferens* (Anderson et al., 1989, 1993; Olsen, 1989; Gobler et al., 2005). Since then, this pelagophyte has been documented annually in Maryland, Virginia, and New Jersey (starting in 1995) (Gastrich et al., 2004; Gobler et al., 2005). More broadly, it has been confirmed that *A. anophagefferens* persists in the United States coastally from Florida to Maine (Anderson et al., 1993; Popels et al., 2003). While its presence is generally not at bloom levels and they are instead found at “background concentrations,” under the right conditions these seed populations may produce future blooms (Popels et al., 2003). We note that another pelagophyte, *Aureoumbra lagunensis* DeYoe et Stockwell, can form blooms throughout the coastline of the Gulf of Mexico (DeYoe et al., 1997; Gobler and Sunda, 2012; Anderson et al., 2021) and as far as the east coast of Florida which are also colloquially referred to as brown tide events (Morris et al., 2018; Lopez et al., 2021): herein “brown tide” will solely reference *A. anophagefferens*.

Brown tides have also been documented outside the USA, including Saldanha Bay, South Africa *ca*. 1997 (Pitcher and Calder, 2000; Probyn et al., 2001; Gobler et al., 2005), and coastal waters of China *ca*. 2009 (Kong et al., 2012; Zhang et al., 2012; Chen et al., 2019). Due to the later appearance of bloom events in Saldanha Bay and the Bohai Sea it was thought that *A. anophagefferens* may have traveled by ballast water contained in ships (Doblin et al., 2004). This was made more plausible by the discovery of a dormant (resting) cell stage (Ma et al., 2020). However, this hypothesis has been complicated by sediment core analyses that provide evidence of a historical presence of *A. anophagefferens* in China’s Bohai Sea of over 1,500 years, and subsequently a confirmation of global distribution similar to other pelagophytes (Tang et al., 2019; Choi et al., 2020). Thus, global initiation of brown tides may be a result of local background populations blooming due to extenuating circumstances rather than being seeded by a foreign source.

Brown tide bloom events are designated HABs due to their negative impacts on local ecological communities, including macrofauna and macroflora. Die-offs in the eelgrass *Zostera marina* have been linked to light attenuation by these HABs (Figure 1C; Cosper et al., 1987; Dennison et al., 1989). Eelgrass beds are majors nurseries and shelters for marine fish and invertebrates, making their demise ecologically devastating (Bricelj and Lonsdale, 1997). Brown tides are detrimental to bivalve species including clams, oysters, mussels, and scallops [*Mercenaria mercenaria*, *Mytilus edulis*, *Argopecten irradians* (Gastrich and Wazniak, 2002; Gobler and Sunda, 2012)]. While the loss of habitat may be significant in the killing of bivalves, inhibition of bivalve feeding by certain *A. anophagefferens* strains implies the presence of a yet unidentified algal toxin (Robbins et al., 2010). Brown tides are economically damaging: an early estimate of annual economic loss in Long Island, New York brown tides based on declines in scallop populations was $3M USD (Kahn and Rochel, 1988), a value that compounded over three decades of presumed annual brown tides would now exceed $100M USD. Outside of the effects on macrofauna and macroflora, an intricate web of interactions occur among *A. anophagefferens* and other microbial organisms (Frazier et al., 2007), including viruses (Sieburth et al., 1988; Figure 1B).

## *Aureococcus anophagefferens* is robust and persistent

Described as a picoplankton with spherical cells of 1.5–2 μm in size with a “golden” color (Sieburth et al., 1988), subsequent observations place these cells at 2–3 μm in a vegetative state (Bricelj and Lonsdale, 1997; Ma et al., 2020). *A. anophagefferens* cells possess a single nucleus, chloroplast, and mitochondria, as well as an exopolysaccharide layer (glycocalyx), of which little of the molecular content is understood (Sieburth et al., 1988). Resting stage cells, or “dormant” cells, generated by growth at 4°C in the absence of light have notable physiological differences compared to their vegetative counterparts and are larger (4–6 μm) in size (Tang et al., 2019; Ma et al., 2020). These resting stage cells are capable of “germination” (returning to normal growth conditions) 10 days after re-entry to 21°C and fresh medium (Ma et al., 2020). Previous work has shown that vegetative *A. anophagefferens* can be stored for at least two weeks in the dark before a return and acclimation to increased temperature and light availability (Doblin et al., 2004; Popels et al., 2007).

Assimilation of carbon and nitrogen are an important component of the *A. anophagefferens* relationship with their environment—especially in the context of a bloom initiation and termination. Of specific note, blooms can occur at points when dissolved organic nitrogen levels are high (Laroche et al., 1997) and when inorganic nutrients are low (Keller and Rice, 1989), as summarized by Gobler et al. (2002). Utilization of many forms of organic nitrogen has been verified in culture (Berg et al., 2002; Gann et al., 2022) and transporters specific for nucleosides, oligopeptides, and purines in the algal genome suggest the capability of diverse nitrogen species assimilation when inorganic nitrogen is depleted (Gobler et al., 2011). Additionally, dissolved organic carbon consumption may also be important during the growth stage of a bloom (Gobler et al., 2002, 2004). Evidence of heterotrophy in *A. anophagefferens via* glucose uptake has been demonstrated in culture (Dzurica et al., 1989; Berg et al., 1997; Lomas et al., 2001). Heterotrophy (making *A. anophagefferens* a mixotroph) likely permits continued growth or population maintenance during a bloom where a species exhibiting exclusively photoautotrophy may not persist. Specifically, light attenuation, and consequently the inability for an *A. anophagefferens* population to meet their requirement for photosynthetic carbon would be counteracted by heterotrophic assimilation (Berg et al., 1997). Other marine algae have also been noted to employ mixotrophy to feed on nitrogen or phosphorus-rich organic matter as a means of obtaining macronutrients (Jeong, 2011; Koppelle et al., 2022), strategies that may also be important for *A. anophagefferens*.

Trace elements have also been tied to successful propagation of *A. anophagefferens*. Of particular importance is selenium, an element required for biosynthesis of the 21st amino acid selenocysteine, which in turn has been identified in 59 proteins encoded by *A. anophagefferens* (Gobler et al., 2011). Increasing selenium concentrations as high as 0.01 nM in culture with *A. anophagefferens* was correlated with increased growth rate and *in situ* dissolved selenium concentration was found to be highest prior to brown tide bloom initiation and lowest during bloom termination (Gobler et al., 2013). Similar to many other HAB-forming species, *A. anophagefferens* is an auxotroph for vitamin B_12_ and biotin (Tang et al., 2010). Likewise, vitamins B_12_ and B_1_ depleted populations of *A. anophagefferens* exhibited increased growth when introduced to vitamin replete conditions, signifying a dependence on external sources of these vitamins (Koch et al., 2013).

## A virus infecting *Aureococcus anophagefferens*

While a virus infecting *A. anophagefferens* was not isolated until the late 1990s, virus-like particles within this brown tide system had been previously noted in detail (Sieburth et al., 1988). During the initial characterization of *A. anophagefferens* in 1985 *via* transmission electron microscopy (TEM), infection by large icosahedral viruses was observed *in vivo*, both before and after the dissolution of most cellular organelles (Sieburth et al., 1988). It was shown that a population of lytic viruses of *A. anophagefferens* were present in brown tide blooms, as lysing *A. anophagefferens* cells were observed expelling viral particles (Sieburth et al., 1988). Infected *A. anophagefferens* was also observed in the food vacuole of phagotrophic grazers (Sieburth et al., 1988).

TEM studies of bloom samples between 1999 and 2002 revealed icosahedral viral-like particles in *A. anophagefferens* cells in coastal New York and New Jersey as early as April and as late as September (Gastrich et al., 2004). The proportion of virally infected cells was shown to vary. Between May and June (the bloom’s peak in cell density), TEM imaging revealed less than 10% of cells were infected at any given time, while in July and onward 20–38% of *A. anophagefferens* were infected (Gastrich et al., 2004). By the last sampling date in September when the bloom had decreased in cell density by over 30-fold, 21.4% of cells contained viral particles (Gastrich et al., 2004). This approach with natural populations of *A. anophagefferens* was the initial driver used to suggest that viral infections were a major factor in collapse of brown tide blooms (Gastrich et al., 2004).

Although infection within natural populations had been visualized, isolation of a virus proved difficult. Originally, a concentrated viral fraction *vis a vis* (Suttle et al., 1991) of brown tide seawater was used to lyse lab-grown cultures of *A. anophagefferens* (Milligan and Cosper, 1994). Electron microscopy of this lysate revealed numerous tailed viral particles resembling bacteriophages (Milligan and Cosper, 1994). Capsids were approximately 60 nm in diameter, and multiple viral particles were shown associated with *A. anophagefferens* at any given time, yet no images of tailed viral particles were found within the cell (Milligan and Cosper, 1994). Further analyses were performed using these isolates to study the life cycle of the virus, which appeared to confirm the ability of a phage-like particle to infect and lyse the eukaryotic host (Garry et al., 1998; Gastrich et al., 1998). However, the presence of heterotrophic bacteria in *A. anophagefferens* cultures (Frazier et al., 2007) and significant morphological discrepancies between the free “phage” and virus particles seen inside infected cells (Gastrich et al., 2004) cast doubt on this claim. Subsequently, it has been suggested that the collapse of host populations from environmental samples were a result of bacteria-induced lysis, as several algolytic bacteria were isolated from bloom water (Frazier et al., 2007). The identification of phage-like particles was instead proposed as an artifact of increased bacteriophage activity due to expanding heterotrophic bacterial populations feeding on organic carbon from dead *A. anophagefferens* cells (Frazier et al., 2007; Gobler et al., 2007).

It was not until 2002 that the virus now known as *K. quantuckense* was isolated from Quantuck Bay, NY, with scanning electron microscopy (SEM) and TEM imaging used to confirm large viral particles were attaching to algal cells and viral particles were produced internally (Rowe et al., 2008). This isolated virus was similar to those seen in cells from brown tide blooms, lacking the tail-like structure described in Milligan and Cosper (1994), and was confirmed to be a lytic agent of *A. anophagefferens* after successive isolations and infections (Rowe et al., 2008). The newly isolated virus was screened for infectivity on a litany of algal species and was found to lyse 8 out of 19 *A. anophagefferens* strains tested and unable to lyse phylogenetically distinct species (Gobler et al., 2007). After numerous attempts to characterize the viruses using PCR-based molecular tools designed for algal viruses (of the order *Algavirales*) (Rowe et al., 2008), the genome of *K. quantuckense* was sequenced in 2014 (and re-sequenced in 2022) to better resolve functional capabilities of the virus by defining the genetic potential (Moniruzzaman et al., 2014; Truchon et al., 2022).

## *Kratosvirus quantuckense* represents a clade of understudied algal viruses

**FIGURE 2:**
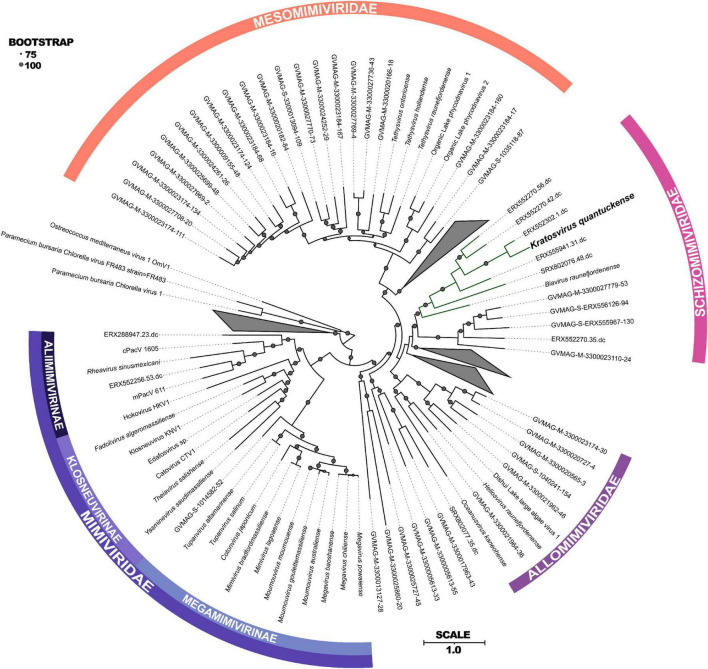
Maximum-likelihood phylogenetic tree constructed with RAxML and visualized using iTOL (Stamatakis, 2014; Letunic and Bork, 2021) of concatenated protein sequences among all *Imitervirales*. Bootstrap values greater than 75 are shown with size corresponding to confidence. Families and subfamilies are detailed by different colors. Distinct clades containing only environmentally sequenced viruses are collapsed. Adapted from Aylward et al. (2023).

Following DNA sequencing, *K. quantuckense* was found to phylogenetically fit into the phylum *Nucleocytoviricota* (Moniruzzaman et al., 2014). These viruses are notable for their size and are associated primarily with infection of eukaryotic organisms, ranging from *Amoebozoa* to humans (Wilhelm et al., 2017). At the time of sequencing, only a handful of viruses collectively known as “giants” were available for study. Since then, the known diversity of *Nucleocytoviricota*, especially among viruses closely related to *K. quantuckense*, has been expanded by metagenome-assembled genomes (MAGs) generated from diverse environments, including aquatic, terrestrial, permafrost, and arthropod-associated derived datasets (Moniruzzaman et al., 2020; Schulz et al., 2020). These studies have revealed a wealth of diversity existing among phylogenetically similar viruses, although for most host information is lacking (Needham et al., 2019). Phylogenomic structuring of the *Nucleocytoviricota* taxonomy using the information provided from MAGs has helped to resolve discrete lineages of viruses previously undefined due to limited viral and host isolation (Aylward et al., 2021). Through concatenation of several core genes a novel phylogeny of the *Imitervirales*, a monophyletic order consisting of all *Mimiviridae* and “extended *Mimiviridae*” (Aylward et al., 2021), was constructed including MAG representatives from publicly available metagenomes (Figure 2).

Phylogenetically, *K. quantuckense* groups with other large double stranded DNA viruses of eukaryotic algae [both in single gene phylogenetic reconstruction and concatenated protein phylogenies, including DNA polymerase B (polB) and the major capsid protein (MCP)], most of which have phototrophic hosts (Figure 2; Coy et al., 2018; Stough et al., 2019). *K. quantuckense* is most closely related to Prymnesium kappa virus (PkV; *Biavirus raunefjordenense*) among all presently isolated viruses (Figure 2; Johannessen et al., 2015). Also closely related are Tetraselmis virus 1 (*Oceanusvirus kaneohense*), Pyramimonas orientalis virus (*Heliosvirus raunefjordenense*), and the proposed *Mesomimiviridae* clade which includes the Organic Lake phycodnaviruses (Sandaa et al., 2001; Yau et al., 2011; Schvarcz and Steward, 2018). With the inclusion of viral MAGs, *K. quantuckense* groups into the recently demarcated family *Schizomimiviridae*, where PkV is the only other cultured representative. This increase in diversity across closely related viruses highlights that isolated viruses only represent a small proportion of all environmental algal viruses.

Of the virus isolates which neighbor *K. quantuckense* (Figure 2), the only one which does not infect a phototroph is a putative choanoflagellate infecting virus, *Mimiviridae* sp. ChoanoV1, identified through single-cell metagenomics (Needham et al., 2019). This alga centric clade of giant viruses shares many common core genes including two DNA-directed RNA polymerase 2 subunits (Rpb2) in each genome (Blanc-Mathieu et al., 2021). Although originally attributed to the *Mesomimiviridae* alone, resequencing of the *K. quantuckense* genome revealed a second Rpb2 gene that had previously not been detected (Truchon et al., 2022). These divergent subunits are likely the result of an ancient gene duplication event in a common ancestor (Blanc-Mathieu et al., 2021). Other *Nucleocytoviricota* outside of this monophyletic clade are known to share this trait: Cafeteria roenbergensis virus (CroV; *Rheavirus sinusmexicani*) also has two copies of this gene, though these have a much higher amino acid identity (> 99%), pointing to a more recent duplication event (Fischer et al., 2010). While DNA-directed RNA polymerases are a conserved genotype among *Nucleocytoviricota* (Sharma et al., 2014), the presence of divergent Rpb2 subunits in these clades may be a defining characteristic of viruses of phototrophic algae. This host-virus system presents a unique opportunity to explore the role of these duplicated genes during infection and to infer conserved characteristics among viruses of other bloom-forming algae.

## The “Little Giant”: structural analysis and cryptic entry

**FIGURE 3:**
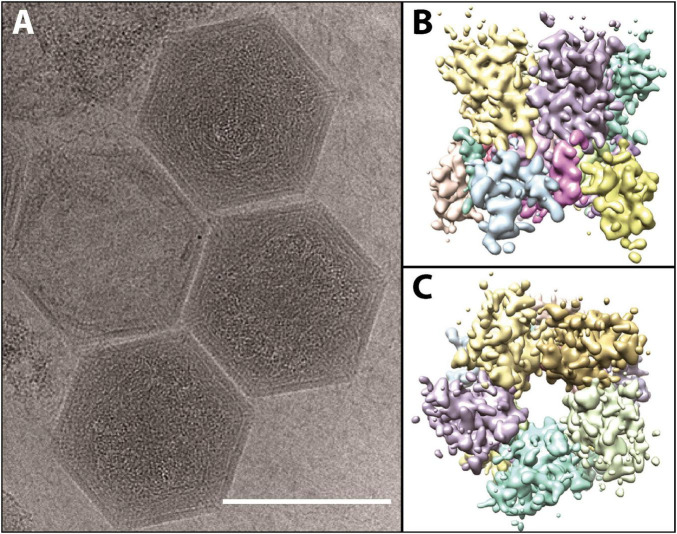
**(A)** Cryo-electron microscopy images of fully formed *K. quantuckense* viral particles (scale bar; 1,500 Å). **(B,C)** Computational reconstruction detailing the side **(B)** and top **(C)** view of the pentomeric capsomer. Images are adapted from work performed in Gann et al. (2020c).

Although taxonomically it is a “giant virus,” *K. quantuckense* has an icosahedral capsid that is approximately 190 nm (Figure 3A; adapted from Gann et al., 2020c), smaller than the capsids of amoebal *Nucleocytoviricota* (Rowe et al., 2008; Legendre et al., 2018) although the same size as Paramecium bursaria Chlorella virus (PBCV-1) which infects the green algae *Chlorella variabilis* (Shao et al., 2022). The capsid is mainly composed of a single MCP trimerizing into a pseudohexagonal capsomer covering most of the capsid surface (Gann et al., 2020c). Pentameric capsomers are located at the vertices of the capsid, though it is unclear which protein(s) form these structures (Gann et al., 2020c; Figures 3B, C). A capsid composed of multiple minor capsid protein subunits alongside the MCP has been described previously (Xiao et al., 2017). The capsid has a triangulation number, or *T* number, of 169, with *h* and *k* values of 7 and 8, respectively (Gann et al., 2020c). Like other *Nucleocytoviricota*, the MCP has a “double-jelly roll” structure, which is very common in icosahedral viruses (Krupovic and Koonin, 2017). However, unlike some other *Nucleocytoviricota*, *K. quantuckense* lacks additional structures extending from the viral capsid (La Scola et al., 2003; Cherrier et al., 2009; Abrahao et al., 2018; Gann et al., 2020c). Additional structures are commonly associated with attaching and eventual entry into the host cell, leaving no such evidence in the case of *K. quantuckense* (Cherrier et al., 2009; Milrot et al., 2017). Without obvious additional surface structures, this necessitates further structural analyses of the viral capsid to identify additional binding mechanisms outside the protein layer.

Similar to other *Nucleocytoviricota*, there is evidence to suggest *K. quantuckense* possesses an internal membrane (Gann et al., 2020c). This is likely involved in fusion between the host cell and the viral cell upon infection. During infection of *Chlorella variabilis* NC64A by PBCV-1, the spike protein initiates contact with the host cell (Cherrier et al., 2009), before the viral membrane fuses with the host membrane and DNA is injected into the cell (Milrot et al., 2017). It is possible that a similar method of infection occurs between *K. quantuckense* and *A. anophagefferens*, in which membrane fusion facilitates the entry of viral DNA into the host. This was implied through early SEM imaging (Rowe et al., 2008). In other cases, such as that of Acanthamoeba polyphaga mimivirus (APMV) where the entire viral particle is taken up into the cell, the internal membrane fuses with a phagosome after the opening of the viruses’ “stargate” portal, a specific vertex on the viral capsid that can undergo a conformational change (Zauberman et al., 2008; Xiao et al., 2009). Like APMV in this case, *K. quantuckense* possesses a pocket beneath vertices of the particle, showing that the internal membrane is not evenly spaced beneath the capsid (Gann et al., 2020c). It is possible that packaged proteins within the particle, of which *K. quantuckense* has 43, are present in these pockets and may be involved in the initiation of infection, as they will be the first proteins to interact with the host (Gann et al., 2020c). But while cryo-electron microscopy has revealed unique vertices in certain Mimiviruses (Xiao et al., 2005, 2017), no such portal structure is yet identified on the *K. quantuckense* capsid (Gann et al., 2020c), thus leaving a missing piece in our understanding of infection in this host-virus system. Still, key similarities with other *Nucleocytoviricota* despite its own unique characteristics make this virus an interesting focal point for studying algal virus infection initiation.

## Evidence of viral genomic streamlining and adaptation

**FIGURE 4:**
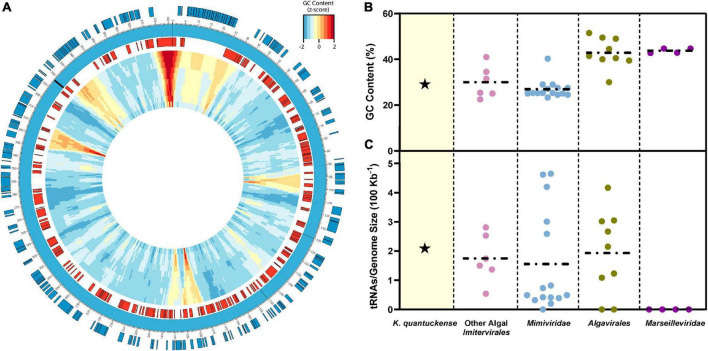
**(A)** Representative genomic map of *K. quantuckense*. Coding sequences on the positive strand are shown in blue and coding sequences on the negative strand are shown in red. Genomic GC-content is displayed in relative z-score via a window moving 500 bp with a size ranging from 10,000 bp to 1,000 bp from the outer ring to the inner ring. tRNAs are labeled on the genome as black bars. **(B)** GC content and **(C)** tRNAs encoded normalized to genome size of *K. quantuckense* as compared to several other taxa of *Nucleocytoviricota*. Horizontal dotted lines depict the mean of each individual group of viruses for GC content and tRNAs.

The genome of *K. quantuckense* has been analyzed to determine coding potential of the virus (Figure 4A). The first assembly of the *K. quantuckense* genome generated from Illumina short reads was 371 kbp with 376 coding regions and eight tRNAs (Moniruzzaman et al., 2014). The virus notably had a highly distinct GC-content compared to the host: 28.7 vs. 69.9% (Gobler et al., 2011; Moniruzzaman et al., 2014; Figure 4B). This increase in adenine/thymine frequency in the virus may suggest utilization of a salvage pathway to convert host nucleotides to nucleotides suitable for the *K. quantuckense* genome, a shift common to viruses of single-celled eukaryotes (Brown and Bidle, 2014; Simón et al., 2021). A high conservation of tRNAs in T4-like *Myoviridae* has further been proposed as a means for maintaining a large disparity in GC-content between virus and host (Limor-Waisberg et al., 2011) and although no such analysis has been observed in *Nucleocytoviricota*, the presence of tRNAs and tRNA synthetases in these genomes may imply an evolutionary specialization for specific hosts (Abergel et al., 2007; Santini et al., 2013; Rodrigues et al., 2019; Figure 4C). *K. quantuckense* appears to favor tRNAs encoding for AT-rich anticodons rather than GC-rich anticodons in most cases. While most of these specific tRNAs are also encoded by the host *A. anophagefferens* CCMP1984 genome, *A. anophagefferens* tRNA anticodons have a GC-content of 51.3% while *K. quantuckense* tRNA anticodons have a GC-content of 25%, possibly pointing to an enrichment in GC-depleted tRNAs during infection.

Recently the virus was re-sequenced using a combination of Oxford Nanopore long-reads and Illumina short-reads, providing a new representative genome which better resolved the repetitive regions of the viral genome (Truchon et al., 2022). Long reads which extended beyond the terminal ends confirmed that this genome is circular instead of linear and contained 384 coding regions, including several duplications of previously annotated genes (Truchon et al., 2022). It is as of yet unclear whether these changes can be explained by evolution of the viral genome through maintenance of the strain during the last 20 years in a laboratory setting or simply higher resolution databases and tools used for predicting genes.

The previously hypothesized “terminal” ends of the genome were characterized by leucine-rich repeats and proteins containing a domain of unknown function (DUF285) (Moniruzzaman et al., 2014). Although the role of these regions is unknown in *K. quantuckense*, they have been implied to be involved in the mobilome, or the entirety of mobile genetic elements incorporated in a genome (Frost et al., 2005), of certain bacteria, and more specifically Mollicutes (Röske et al., 2010). Based on the new resequencing data, the viral genome appears to be divided into a repetitive DUF285-rich region representing approximately two thirds of the genome and a non-repetitive region dense in coding potential and conserved *Nucleocytoviricota* orthologs (Moniruzzaman et al., 2014).

Of the *Nucleocytoviricota* infecting unicellular eukaryotes, *K. quantuckense* has a relatively small genome, rivaled by the similarly sized PBCV-1 and the 180 kbp genomes of the Prasinoviruses (Van Etten et al., 1991; Derelle et al., 2008; Moreau et al., 2010; Moniruzzaman et al., 2014). Genome streamlining is common among obligately intracellular life forms, both viruses and bacteria alike (Luo et al., 2011; Leobold et al., 2018). One can imagine the expansion of a virus pangenome to permit infection of variable hosts and strains, and a cyclic removal of extraneous genes as host-virus co-evolution occurs [e.g., the Red Queen effect (Elde et al., 2012)]. However, a noted difference between the newly sequenced genome and the formerly sequenced genome is the apparent duplication of genes (Truchon et al., 2022). This was previously hypothesized to have occurred within the DUF285 regions of the virus and would serve the opposite function of genomic streamlining (Moniruzzaman et al., 2014). The role of these duplication events and how frequently they occur remains unclear, but their presence points to some functional significance that better improves the fitness of the virus. In poxviruses, gene duplication events trigger a phenomenon described as a “genomic accordion” mechanism, in which expansion and loss of coding regions at the terminal ends of the genome occurs, posited to encourage rapid evolution of these genes (Elde et al., 2012). Further investigation into environmental strains of *K. quantuckense* or new isolates compared to the current laboratory isolate may reveal heterogeneity among viruses, elucidating evolutionary history and differences in fitness.

## *Kratosvirus quantuckense* encodes a dense genetic repertoire

Within the genome, the coding regions of *K. quantuckense* that can be functionally characterized represent a diverse repertoire of coding potential. Only 123 of the 384 potential coding regions have putative functions, 38 of which are DUF285 containing proteins (Moniruzzaman et al., 2014). Only 16 of the 384 coding regions have an associated annotation in widely used protein functional databases [i.e., Kyoto Encyclopedia of Genes and Genomes (KEGG)]. These genes, and over 100 more, possibly originated from instances of horizontal gene transfer, as they bear more similarity to bacteria (72 genes) and eukaryotes (56 genes) than other viral representatives based on best reciprocal blast hit (Moniruzzaman et al., 2014). Similarly to the bloom-terminating Emiliania huxleyi virus and the HAB-terminating Heterosigma akashiwo virus, *K. quantuckense* has very few conserved orthologs with other frequently studied *Nucleocytoviricota* (Maruyama and Ueki, 2016). Even when compared to phylogenetically similar viruses, including PkV, Phaeocystis globosa virus, and Chrysochromulina ericina virus, only a fraction of protein-coding genes are conserved (Gallot-Lavallee et al., 2015, 2017). Between these three viruses there are 49 conserved homologs, and when two more divergent *Mimiviridae* isolates are included in this genomic analysis, *K. quantuckense* still encodes for 274 unique genes (71.35%) (Gallot-Lavallee et al., 2017).

Genetic elements in *K. quantuckense* similar to those in other *Nucleocytoviricota* include machinery for DNA replication, transcription, and translation (Yutin et al., 2009). The virus encodes for a litany of RNA polymerase subunits and core *Nucleocytoviricota* replication genes including DNA polymerase B, a topoisomerase, helicase, and a DNA photolyase (Moniruzzaman et al., 2014). And while many *Nucleocytoviricota* lack translation-specific machinery, *K. quantuckense* encodes a eukaryotic translation initiator-like protein and a translation elongation factor. Also encoded are proteins likely involved in the process of generating translatable mRNA through nucleotide modification (e.g., ubiquitin transferases and an mRNA capping protein). The presence of these and similar eukaryote-like genes may highlight the evolutionary past of the virus with a history of horizontal gene transfer, as while they are conserved between other similar algal viruses, they are holistically different from translational machinery in other *Nucleocytoviricota* (Jeudy et al., 2012; Moniruzzaman et al., 2014).

The viral genome also possesses three coding sequences which bear no similarity to other known viral proteins. These encode for putative pectate lyases, proteins involved in the degradation of complex plant tissue (Moniruzzaman et al., 2014). These proteins do not appear to be acquired from their host as determined by phylogeny (Gann et al., 2020c) but instead potentially bacterial in origin based on sequence homology. Polysaccharide degrading enzymes are not unique to this system, as one was recently isolated from the PBCV-1 viral particle and was shown to digest the cell wall of non-specific *Chlorella* hosts (Agarkova et al., 2021). While *A. anophagefferens* cells lack a cell wall, the dissolution of the glycocalyx during infection occurs both in natural blooming populations and in culture (Sieburth et al., 1988; Rowe et al., 2008). It should be noted, however, that *A. anophagefferens* does encode for phylogenetically distinct pectate lyases, which is typically uncommon in eukaryotes (Gobler et al., 2011). These viral genes could also serve to maintain metabolic functions for required nutritional needs after host machinery has been broken down through infection. Whether these pectate lyases are engaged in this function remains to be seen, but their prevalence in the genome implies an important function.

Like the pectate lyase genes, there are five homologous putative concanavalin A-like lectin/glucanases (Moniruzzaman et al., 2014). This orthologous group consists of genes encoding proteins which either bind or break down complex polysaccharides (Burgess et al., 2009). This superfamily includes proteins that degrade carbohydrates of algae and plants including xylan and alginate. Various algae are also a source of lectin/glucanases used in biomedical research and three strains of *A. anophagefferens* encode for a potential lectin/glucanase as well (Gann et al., 2022). This may highlight a point of origin for the presence of these lectin/glucanases in the viral genome. There appears to be a conserved role in polysaccharide binding and degradation within the viral genome, given the encoded three putative pectate lyases and the five putative lectin/glucanases. While carbohydrate metabolism genes are common in *Nucleocytoviricota*, in many cases they are primarily glycosyltransferases (Table 2; Van Etten et al., 2017). Such a high frequency of non-glycosyltransferase carbohydrate metabolism genes in *K. quantuckense* implies an important role of at least some of these proteins in infection. Notably, one pectate lyase and three lectin/glucanases were packaged within the viral particle, underscoring their importance and effectively making the viral particle capable of these metabolic processes immediately upon host invasion (Gann et al., 2020c). Molecular cloning and purification of some of these proteins of interest is vital to further understanding the mechanisms of the *K. quantuckense* infection cycle.

**TABLE 2:** Carbohydrate metabolism (CM) genes encoded by *Kratosvirus quantuckense* and various other *Nucleocytoviricota*.

Virus	CM Genes	Glycosyltransferases	Polysaccharide lyases	CM genes/genome Size (Mbp)	Notes
***Imitervirales***					
*Kratosvirus quantuckense*	8	4	3	20.779	Pectate lyase-like
*Tethysvirus hollandense*	6	6	0	13.044	
*Tethysvirus raunefjordenense*	11	10	0	23.228	
*Oceanusvirus kaneohense*	5	3	0	7.485	
*Rheavirus sinusmexicani*	3	3	0	4.859	
*Theiavirus salishense*	6	5	0	4.329	
*Cotonvirus japonicum*	10	9	0	6.773	
*Tupanvirus altamarinense*	18	12	0	12.504	
*Tupanvirus salinum*	19	13	0	12.531	
*Megavirus chilense*	8	8	0	6.353	
*Mimivirus bradfordmassiliense*	13	12	0	11.003	
*Moumouvirus moumou*	9	9	0	8.812	
***Algavirales***					
Micromonas pusilla virus SP1	4	4	0	23.061	
Ostreococcus tauri virus OtV5	8	6	0	42.847	
Ostreococcus lucimarinus virus OlV5	8	7	0	43.882	
Paramecium bursaria Chlorella virus 1	16	9	2	48.395	Alginate lyase-like
Acanthocystis turfacea Chlorella virus 1	12	6	2	41.659	Glucoronan/ alginate lyase-like
Ectocarpus siliculosus virus 1	2	1	0	5.960	
Emiliania huxleyi virus 86	1	1	0	2.455	
***Pimascovirales***					
*Tunisvirus*	2	2	0	5.263	
*Lausannevirus*	2	2	0	5.768	
*Marseillevirus marseillevirus*	2	2	0	5.428	
**Other**					
*Pandoravirus kuranda*	2	1	0	1.048	
*Pithovirus sibericum*	3	3	0	4.918	
*Mollivirus sibericum*	1	1	0	1.535	

Annotations of metabolism genes, glycosyltransferases, and polysaccharide lyases are based on the Carbohydrate Active Enzymes (CAZy) database (Drula et al., 2021).

There were eight putative nucleic acid methyltransferases encoded by the virus in the initial annotation of *K. quantuckense*. While one is likely associated with the mRNA capping process, the other seven are predicted to methylate viral or host DNA: five of these have been predicted by the gold-standard database REBASE (Roberts et al., 2022). While the targeted motif for these proteins remains unknown, DNA methylation is not uncommon among giant viruses (Jeudy et al., 2020). While certain *Nucleocytoviricota* do not have any detectable methylation in their genomes despite encoding methyltransferases, others have been shown to methylate their own DNA (Jeudy et al., 2020). As an example, PBCV-1 encodes for several adenine-specific methyltransferases, as well as the tandem restriction endonucleases (Xia and Van Etten, 1986; Zhang et al., 1998; Coy et al., 2020). Likewise, Phaeocystis globosa virus (PgV) encodes for several methyltransferases, only one of which shares high homology to genes encoded by distinct lineages of Mimiviruses (Santini et al., 2013). These are likely important for protecting viral DNA from digestion by the virus’ own endonucleases, which are used to target the host genome (Chan et al., 2006) and significantly degrade the host DNA within 60 min of infection (Agarkova et al., 2006). It has been shown in iridoviruses that methylation of viral DNA is associated with the generation of a larger viral progeny (Goorha et al., 1984).

The role of methylation in *K. quantuckense* requires further examination, as it is unclear whether it is required for the propagation of the viral progeny. The virus does encode a relatively higher number of methyltransferases compared to other *Nucleocytoviricota* (Coy et al., 2020). DNA methylation may also serve as a means for controlling expression of viral genes in different stages, as temporal gene expression is an important element of the infection cycle. Ultimately, the uniqueness of the *K. quantuckense* coding potential makes it an interesting focal point of study that might better define similar uncultured viruses.

## The viral transcriptome and proteome signify robust host takeover capabilities

To better understand the infection cycle of *K. quantuckense* and how the host responds to viral invasion, transcriptomic profiling was performed following inoculation of *A. anophagefferens* CCMP1984 with virus (Moniruzzaman et al., 2018). Transcription of *K. quantuckense* genes was observed within 5 min, and although there were very few viral reads at this time point, the virus accounted for 50% of all transcripts by the 21-h time point (Moniruzzaman et al., 2018).

Host transcriptional responses rapidly changed following infection with *K. quantuckense*, with over one thousand genes being differentially expressed 5 min following infection (Moniruzzaman et al., 2018). This rapid shift in expression patterns following infection is not unique to this system, but temporal transcriptomes of *Nucleocytoviricota* infections are sparse, highlighting a gap in our understanding of these viruses (Blanc et al., 2014; Ku et al., 2020). Twelve hours after infection, 43% of *A. anophagefferens* host genes were differentially expressed, a pattern which continued as the infection progresses (Moniruzzaman et al., 2018). It is likely that the initial shift in host gene expression is driven by an immediate response to infection, potentially as a means of defense, before the virocell develops.

Almost one third of all viral genes were expressed within the first 5 min of infection (Moniruzzaman et al., 2018). By the final time point only three genes had gone undetected in the transcriptome (Moniruzzaman et al., 2018). Given a temporal pattern of gene expression had been detected like other *Nucleocytoviricota*, coding regions of the genome were examined for early and late promoters (Legendre et al., 2010; Blanc et al., 2014; Rodrigues et al., 2020). While no significant promoters were detected for genes expressed later in the infection cycle, a motif conferring early transcription was enriched among genes expressed within the first 6 h of infection (Moniruzzaman et al., 2018). Notable genes expressed early in the infection cycle were a combination of methyltransferases, carbohydrate metabolism-associated genes, proteases, and transcription factors. While genes exclusively expressed in the latent stage of infection (between 12 and 21 h) were largely associated with virion structure and composition, the MCP was expressed immediately and constitutively throughout infection, making up over half of all viral reads by the 21-h time point (Moniruzzaman et al., 2018). This is a unique element in the *K. quantuckense* infection cycle, as in many previously described *Nucleocytoviricota* structural components are not expressed until later stages of infection (Blanc et al., 2014; Rodrigues et al., 2020). It has been noted, however, that MCP transcripts are the most readily detectable *Nucleocytoviricota* elements across multiple species in a bloom setting (Moniruzzaman et al., 2016). Infection patterns in other *Nucleocytoviricota* entail early genes being “shut off” later in transcription and “host takeover” genes being forgone for auxiliary metabolic genes and structural components (Blanc et al., 2014; Rodrigues et al., 2020). However, in *K. quantuckense* all viral genes increase in expression throughout infection (Moniruzzaman et al., 2018). This unique pattern highlights the diversity of infection cycles across the *Nucleocytoviricota* and even across algal viruses, further justifying the importance of introducing new model systems such as this one for holistically understanding marine viral infection dynamics.

Evidence for “repurposing” of the host machinery has been identified in the *A. anophagefferens* and *K. quantuckense* host-virus system as well as other *Nucleocytoviricota*. This includes an early suppression of cytoskeleton and fatty acid metabolism and increased expression of transcription regulation genes (Moniruzzaman et al., 2018). Later in the infection cycle, selenocysteine-containing proteins, translational machinery, and polyamine biosynthesis genes were overexpressed (Moniruzzaman et al., 2018). The overexpression of phosphate transporters may also implicate a need for extracellular phosphorus by the virus (Gann et al., 2020c). However, it has been noted that these transporters could potentially be used for acquisition of selenium (a micronutrient required by the host) as well (Lazard et al., 2010). Initially following infection, expression of photosynthesis-related genes was significantly decreased (Moniruzzaman et al., 2018). This transcriptional shift has been proposed as a host defense mechanism, to both reduce energy availability for viral production and to increase the presence of photosynthetic derivatives like porphyrins, which may make the host cellular environment unsuitable for viruses (Moniruzzaman et al., 2018). Still, the sheer volume of differentially expressed host genes in infected *A. anophagefferens* cells implies more than simply a host reacting to a foreign agent, and instead the colloquial “take-over” associated with viral infection in eukaryotes.

While the earliest detectable transcripts are important to “takeover” of the host cell, the first viral elements to initiate infection are those that are packaged as proteins inside the viral particle. Apart from the MCP, proteomics conducted on the viral particle has revealed 42 proteins present in the intact virus particle (Gann et al., 2020c). As stated, there is an enrichment of polysaccharide degrading enzymes packaged in the capsid (Gann et al., 2020c). Likewise, early expression of viral genes and initiation of the infection cycle after 5 min is likely the result of *K. quantuckense* packaging six transcription related proteins (Gann et al., 2020c). The number of packaged proteins of *Nucleocytoviricota* is correlated to the size of the viral particle and genome (Renesto et al., 2006; Gann et al., 2020c). Still, other *Nucleocytoviricota* of similar capsid diameters package more transcriptional machinery, meaning *K. quantuckense* may be relatively more reliant on the host cell for beginning its infection cycle (Fischer et al., 2014; Abrahao et al., 2018) though packaging of viral mRNA has also been shown to occur in similar viruses (Boyer et al., 2009). PBCV-1 must translocate its DNA into the host’s nucleus in order to access host RNA polymerases, which may be closer to what *K. quantuckense* does rather than packaging all necessary transcriptional machinery like certain Mimiviruses (Fischer et al., 2014; Milrot et al., 2017). Despite this, *K. quantuckense* and other *Mimiviridae* cluster with the Marseilleviruses based on best reciprocal blast hits of the proteome rather than the *Algavirales*, making the *K. quantuckense* proteome an intriguing point of study as it represents one of the only algal virus proteomes of its kind (Gann et al., 2020c).

## *Kratosvirus quantuckense* infection drives morphological shifts in the virocell

**FIGURE 5:**
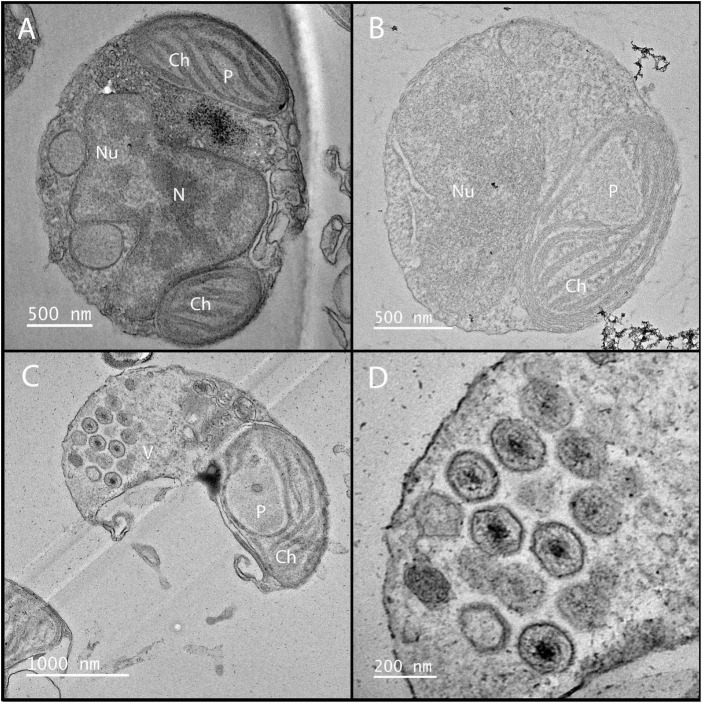
Electron microscopy of *K. quantuckense* showing early stages of infection **(B)** and formation of viral particles **(C,D)** inside a host *A. anophagefferens* cell as compared to a healthy host **(A)**. Nu, nucleus; N, nucleolus; Ch, chloroplast; P, pyrenoid; V, viroplasm.

Following infection *A. anophagefferens* undergoes physiological alterations as it becomes a virocell: a cell undergoing lytic infection that is morphologically, transcriptionally, and metabolically distinct from a healthy host cell (Forterre, 2011). This occurs rapidly after infection, as the electron density of the *A. anophagefferens* cytoplasm increases significantly (Gastrich et al., 2002, 2004). Stark changes in host cell structure are common among *Nucleocytoviricota* infections and can be identified relatively quickly after initiation (Yoshikawa et al., 2019; Figure 5). Upon infection of *A. anophagefferens*, the polysaccharide-rich glycocalyx disappears, pointing to a degradation occurring possibly via viral enzymatic processes (Sieburth et al., 1988; Rowe et al., 2008; Gann et al., 2020c). As this layer will be the first thing the virus interacts with and its preservation may confer resistance to viral infection, the glycocalyx structure remains an interesting focal point of research to further develop our understanding of this host-virus system. This process also mirrors the initial degradation of the cell wall upon infection in other algae infected with *Nucleocytoviricota* (Meints et al., 1984; Van Etten et al., 2017; Agarkova et al., 2021; Table 2).

Infection of single-celled eukaryotes by a giant virus, such as in the amoeba-infecting *Mimiviridae*, typically causes the formation of a viral factory, an organelle-associated structure in the cytoplasm devoted to viral genome replication and capsid construction (Suzan-Monti et al., 2007; Abrahão et al., 2014). Although there is no evidence of these forming in *K. quantuckense*, an electron-dense inclusion, which is the apparent location of viral assembly, forms near the host nucleus, appearing as a separate, unique compartment (Gastrich et al., 1998), similarly to that of Heterocapsa circularisquama virus (Nagasaki et al., 2003). *In situ* analyses of virus infected *A. anophagefferens* have shown that particles are visible while the nucleus is still intact (Sieburth et al., 1988). As virus-like particles begin to form, the host nucleus and other organelles disappear, pointing to the virus repurposing cellular materials (e.g., nucleotide and lipid salvaging) for replicating its own genome (Gastrich et al., 1998, 2002; Figure 5B). During the final stages of infection, the chloroplast is the only remaining organelle (Gastrich et al., 1998, 2002; Gobler et al., 2007; Figure 5C). Chloroplast persistence during *Nucleocytoviricota* infection has been observed in other algae, though often additional organelles are also noted to persist up until lysis (Derelle et al., 2008; Johannessen et al., 2015; Mirza et al., 2015; Pagarete et al., 2015). This suggests a method for the virocell to continue photosynthesizing after infection, though light harvesting complexes are globally down-regulated (Moniruzzaman et al., 2018). Furthermore, a decrease of *in situ* fluorescence (effectively chlorophyll per cell) of late stage infected *A. anophagefferens* cells has been noted (Gann et al., 2020b). While these factors may indicate a decrease in photosynthetic efficiency of the infected cell, the retention of the chloroplast may be a sign of the importance of light to the infection cycle. This is not unheard of, as in certain viruses of cyanobacteria, virally encoded photosystem II genes are thought to be required for replication of the viral genome (Lindell et al., 2005), an important facet as cyanophages are known for degrading nutrient-rich host phycobilisomes (Yoshida-Takashima et al., 2012).

Eventually, virus-like particles fill the entirety of the virocell in a uniform fashion, forming a lattice, leaving only space enough for the undegraded chloroplast (Gastrich et al., 2002; Gobler et al., 2007; Figures 5C, D). The infection cycle is completed after approximately 24 h, at which point host cells lyse and release virus particles, with burst sizes estimated to be between 400 and 500 virions per cell (Rowe et al., 2008; Brown and Bidle, 2014; Gann et al., 2020b). Free viral particles have been identified at the 21 h time point, though these particles may be present as a result of host cells beginning to lyse and not egress from a still intact host cell (Brown and Bidle, 2014). Our understanding of the *K. quantuckense* virocell provides context toward viral dynamics in similar hosts to *A. anophagefferens* and may be used to further elucidate how many cells are infected at a given time point in a bloom. Characterization of the virocell in this system and others is of critical importance to understanding metabolic potential of *in situ* algal populations and the factors that lead to bloom demise.

## Abiotic factors shape infection dynamics

The efficiency of *K. quantuckense* infection is shaped by the factors constraining propagation of the host cell i.e., the intracellular and extracellular environments of the virocell (Gann et al., 2020b). Ecologically, a higher natural availability of macronutrients (inorganic phosphorus and nitrogen) and micronutrients (silicates) correlated to an increase in viral species richness, showing that propagation of specific *Nucleocytoviricota* families depends on external environmental conditions (Clerissi et al., 2014). Under different sources and decreased concentrations of dissolved nitrogen *A. anophagefferens* undergoes significant transcriptional shifts (Frischkorn et al., 2014). Reduced growth of *A. anophagefferens* during nutrient stress could theoretically limit the viral burst size as production of viral particles relies on the availability of nutrients in a cell (Berg et al., 1997, 2008; Gobler et al., 2004). It has been found that a 100-fold reduction in inorganic nitrogen concentration decreased burst size by ∼50% (Gann et al., 2020b). Such a phenomenon was observed in other host-virus systems, with a decreased burst size and increased length of infection cycle (Cheng et al., 2015; Maat and Brussaard, 2016). This was also shown to be a direct result of internal nitrogen within the host cell, as altering nitrogen concentration in the media following infection had no effect on burst size (Gann et al., 2020b).

Virus production has also been verified as being tied to light availability for *A. anophagefferens* (Gobler et al., 2007; Gann et al., 2020a). A decrease in the production of viruses and delay in viral lysis has been noted when *A. anophagefferens* was grown at lower light levels (Gobler et al., 2007; Gann et al., 2020a). This corresponded with a decreased host growth rate, where doubling time shifted from 1.39 to 1.76 days under low light (Gann et al., 2020a). This decrease in growth rate is likely what causes a decrease in viral burst size when cells are infected under lower light. Energy stored in algal cells acclimated to high irradiance levels leads to increased viral lysis of cells in the dark, pointing to viral reliance on photosynthetic-driven generation of ATP (Baudoux and Brussaard, 2008). Although data suggests *A. anophagefferens* can grow heterotrophically, at least for short periods (Popels et al., 2007), it appears that infection and the production of new virus particles requires energy and metabolites associated with phototrophic growth. Similar findings have been described during infection of the bloom-forming *Micromonas pusilla* by Micromonas pusilla virus (Brown et al., 2007).

**FIGURE 6:**
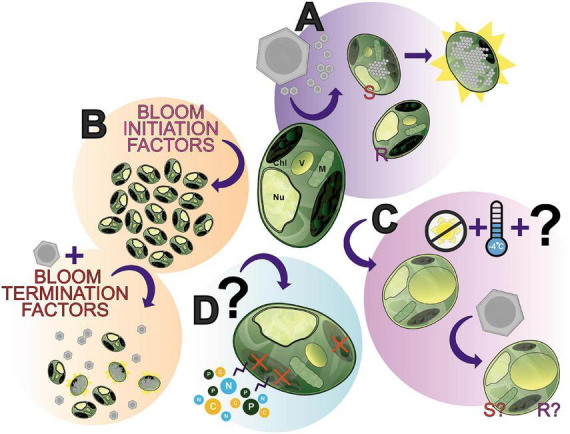
The many “states” of *Aureococcus anophagefferens*; a showcase of what makes this alga an interesting study species and where future research can be directed. **(A)** *A. anophagefferens* forms a host-virus system with *K. quantuckense* with the inclusion of both resistant (R) and susceptible (S) strains of *A. anophagefferens* readily available for experimentation. **(B)** A complex of bloom initiation factors produce harmful algae blooms (HABs) of *A. anophagefferens in situ*. **(C)** An understudied apparent “resting stage cell” or dormant cell has been documented, seemingly the result of low temperatures and the absence of light, thereby producing a cell with unique properties. **(D)** Early studies suggested that under certain conditions *A. anophagefferens* switches to primarily heterotrophy, another understudied aspect of *A. anophagefferens*. Chl, chloroplast; V, vacuole; M, mitochondrion; Nu, nucleus.

Publicly available transcriptomes detailing *A. anophagefferens* gene expression under high (100 μmol m^–2^ s^–1^) and low (30 μmol m^–2^ s^–1^) light (Frischkorn et al., 2014) have been compared to a viral infection transcriptome (Moniruzzaman et al., 2018). The strongest inverse correlation between the low light and infected transcriptomes consisted of genes tied to ribosome biogenesis (Gann et al., 2020a). Decrease in available ribosomal machinery in *A. anophagefferens* under low light may impact the production of viral proteins necessary for the culmination of the infection cycle (Frischkorn et al., 2014; Gann et al., 2020a). As previously stated, pelagophytes have been shown to enter a non-vegetative resting stage (Kang et al., 2017; Ma et al., 2020). As decreases in temperature and irradiance increase the length of infection (Gobler et al., 2007), this resting stage may confer resistance to infection which may ultimately associate the success of infection with the energy partitioning of the host. Such requirements for photosynthetic processes could explain the generation of virally encoded proteorhodopsins found in other *Nucleocytoviricota*, like the HAB-terminating PgV (Yutin and Koonin, 2012). Importantly, differences in susceptibility to infection based on temperature has been characterized in other HAB-forming algae (Nagasaki et al., 2003). Still, infection under these conditions has not yet been properly defined. The spatial positioning of *A. anophagefferens* in the water column should be further analyzed to reveal whether infected cells sink to colder, darker depths as a means of resisting or delaying the effects of infection (Figure 6C).

Other factors, like temperature, are theorized to impact viral propagation as well. In cyanobacteria, infection by phage has been shown to depend on the availability of phosphorus to the host cell (Wilson et al., 1996). External phosphorus concentration, particularly the ratio of nitrogen to phosphorus, is also implicated in *A. anophagefferens* growth dynamics, as shown through differential expression of algal genes under different phosphorus concentrations (Wurch et al., 2019; Gann et al., 2020b). Over expression of phosphate transporters in the infected host point to the virus repurposing host metabolism to increase the amount of phosphorus in the cell (Moniruzzaman et al., 2018). Often virocells display increased expression of transporters relative to an uninfected host cell, implicating a transition in the priority of host cell growth to nutrient acquisition for viral production (Monier et al., 2017; Waldbauer et al., 2019). Availability of phosphorus, both internal and external, in theory should be directly correlated to the burst size of the virus (Gann et al., 2020b). Burst efficiency depending on phosphorus has been described in other viruses of bloom-forming algae, particularly Micromonas pusilla virus and PgV (Maat et al., 2016a,b).

## *Kratosvirus quantuckense* on a global scale: an active contributor

Ecologically, *K. quantuckense* and phylogenetically similar viruses are pertinent in *A. anophagefferens* bloom progression. Amplification of *Mimiviridae* MCP via PCR revealed *K. quantuckense* and a highly similar (87% amino acid identity) virus were among the most abundant viruses in the early stages of the bloom (Moniruzzaman et al., 2016). Furthermore, mapping metatranscriptomic reads from brown tide blooms to *K. quantuckense* reveals that normalized *K. quantuckense* read count follows the trajectory of the bloom itself, collapsing in frequency at the same time *A. anophagefferens* reads disappear (Gann et al., 2021). While the total viral community was highly diverse throughout the bloom, eukaryotic DNA viruses shifted from being less abundant than bacterial DNA viruses following bloom collapse (Gann et al., 2021). Specifically, changes in the community of *Mimiviridae* in brown tide blooms in multiple locations was heavily associated with the progression of the blooms from peak abundance to collapse (Moniruzzaman et al., 2016). This points to the rise of diverse bacteria that colonize bloom waters in the absence of *A. anophagefferens*, though *A. anophagefferens* can cohabitate the water column with other microbes, namely diatoms and cyanobacteria (Gobler et al., 2011; Gann et al., 2021). A co-occurrence network analysis of brown tide metatranscriptomes revealed activity of diatoms, other pelagophytes, other *Mimiviridae*, and ssDNA viruses associated with *K. quantuckense* and *A. anophagefferens* (Moniruzzaman et al., 2017). Thus, we see a diverse community of host-virus pairs in which the infection and nutrient cycling dynamics of one pair may dictate the succession of another.

The role of *K. quantuckense* in *A. anophagefferens* blooms has been defined around North America to an extent, but its ecological role on a global scale has gone largely uncharacterized. While there is a lack of sequenced *Pelagophyceae* genomes available and very few type representatives from cultured sources, these algae are globally distributed at the marine deep chlorophyll maximum (Worden et al., 2012; Choi et al., 2020) and exhibit extremely diverse life histories (Wetherbee et al., 2021; Freyria et al., 2022). Thus, it stands to reason that viral infection of pelagophytes may be an important driver of community composition in other contexts than bloom formation. TARA Oceans-derived metagenomes were analyzed for polysaccharide lyases of viral origin, five of which were clustered with *K. quantuckense* putative pectate lyases (Gann et al., 2020c). Interestingly, of the five putative viruses originating from three sampling sites, four were from the South Atlantic Ocean off the coast of Cape Town, South Africa (Gann et al., 2020c). As *A. anophagefferens* blooms have been documented in coastal water of South Africa (Probyn et al., 2001), this may implicate *K. quantuckense*-like viruses as significant factors in brown tide blooms at other locations world-wide. Other -omics studies have revealed *K. quantuckense*-like viruses in different locations globally (Endo et al., 2020; Gao et al., 2021). Putative viral metagenomic sequences closely related to *K. quantuckense* were identified at approximately 2% of the viral population in the North Atlantic and Arctic Oceans, despite a low abundance of *Pelagophyceae* (Gao et al., 2021). *Nucleocytoviricota* polB sequences from TARA Oceans metagenomes revealed an enriched clade of *K. quantuckense*-like viruses in non-polar regions (Endo et al., 2020). One study based on construction of high confidence marine *Nucleocytoviricota* MAGs from publicly available worldwide metagenomes identified 41 novel MAGs in a clade where *K. quantuckense* was the only isolated representative (Moniruzzaman et al., 2020). Another similar study identified 50 marine *K. quantuckense*-like MAGs, along with several identified in freshwater environments (Schulz et al., 2020). This information implies that there is a wealth of diversity of *K. quantuckense*-like viruses in Earth’s oceans, as they consistently appear in -omics datasets. While it is unclear whether these uncultured viruses infect other strains of *A. anophagefferens* or even other pelagophytes, their presence in coastal blooms and other marine ecosystems suggests they play an important role in biogeochemical nutrient cycling and further validates the use of *A. anophagefferens* and *K. quantuckense* as a relevant model system for heretofore uncultured yet ecologically relevant *Nucleocytoviricota*.

## Mechanisms of resistance to infection on a pangenome scale: a knowledge gap

Several strains of *A. anophagefferens* are resistant to infection by *K. quantuckense*, however, mechanisms of resistance to viral infection in eukaryotic algae are largely undescribed save for a few tractable examples (Thomas et al., 2011). Only two strains of *A. anophagefferens* have been shown to be completely susceptible to *K. quantuckense* infection: the frequently studied *A. anophagefferens* CCMP1984 and the less studied *A. anophagefferens* CCMP1851 (Brown and Bidle, 2014). Other strains of the pelagophyte often used in host-centric research are not lysed when inoculated (Brown and Bidle, 2014). Adsorption assays have revealed that some of these resistant strains are completely resistant to virus attachment as well (Brown and Bidle, 2014). Given the disintegration of the glycocalyx occurs early during successful infection, membrane and extracellular components of different strains may affect virion binding, though any metabolic differences between strains are unclear.

Inhibition of viral adsorption is not the only proposed resistance mechanism against viral infection. *A. anophagefferens* CCMP1848 shows a loss in cell density due to viral inoculation, but this does not lead to complete lysis of the culture or production of viral particles (Brown and Bidle, 2014). Gobler et al. (2007) reported a similar outcome for strains CCMP1850 and 1852. This could mean that while *K. quantuckense* can invade the host cells, a functional defense response by the host could provide resistance to viral production, as has been described in other algal infection studies (Feldmesser et al., 2021). With so little known about the mechanism of viral entrance to the host, this system and the tools that have been developed (Table 1) facilitates further research into host-virus binding specificity and adsorption of the virus, with a particular focus on metabolites of infected, uninfected, and resistant cells.

Many viruses with multiple strains including PBCV-1, Emiliania huxleyi virus, Ostreococcus tauri virus, and Heterosigma akashiwo virus display differential infection patterns on variable hosts (Clerissi et al., 2012; Quispe et al., 2017; Feldmesser et al., 2021; Funaoka et al., 2023). Likewise, toxin production has been described as a mechanism for resistance in the HAB-forming dinoflagellate *Heterosigma circularisquama* against ssRNA virus infection (Tomaru et al., 2009). The lone isolated strain of *K. quantuckense* remains the only virus known to infect *A. anophagefferens* to date, but co-occurrence networks suggest similar strains are present in the environment (Moniruzzaman et al., 2016). Therefore, resistance to viral infection may be widespread and diverse in the context of *in situ* algal blooms in the context of all types of viruses, which should be investigated more holistically in future studies. Consequently, the read mapping of environmental sequences to newly sequenced *A. anophagefferens* genomes shows that blooms are a consortium of closely-related strains, suggesting circumventing viral infection may be a strong evolutionary driver of defense mechanisms between strains (Gann et al., 2022). Furthermore, up to 37% of *A. anophagefferens* cells show visible signs of infection in a bloom (Gastrich et al., 2004), possibly implying infection of other strains of *A. anophagefferens* and thus heterogeneity in the viral population. One possible explanation for the persistence of *A. anophagefferens* following bloom-wide infection is the presence of *Lavidaviridae*-like virophages, which have been described and are predicted to allow for the recovery of the algal host through inhibition of its infecting virus (Yau et al., 2011; Fischer, 2021). Isolation and genome sequencing of novel virus strains as well as the development and cryopreservation of lab viral samples under evolutionary pressure could be effective in improving our understanding of these mechanisms. On the level of individual cells, single-cell transcriptomics may prove fruitful in determining how *A. anophagefferens in situ* and *in vitro* reacts to infection (Ku et al., 2020).

## Conclusion

We have presented here an overview of the *A. anophagefferens*—*K. quantuckense* host-virus system due to its stark differences from other thoroughly studied *Nucleocytoviricota* isolates. This system represents a unique opportunity of research potential where many foundational components of research have been addressed, yet interesting questions and gaps in our knowledge for immediate study persist. Viral infection (Figure 6A) has been characterized and transcriptomic data is available, however, many intricacies of the system have not yet been elucidated, such as the exact mechanism of viral particle entry at the commencement of infection or even high-resolution microscopy over the course of infection. Furthermore, continued -omics based analyses of the virus have provided a higher resolution perspective into the capabilities of the virus and its evolution, specifically the function of unique proteins and those relevant to other *Nucleocytoviricota* host-virus systems. Ultimately, the molecular databases of genomes, transcriptomes, and proteomes provide the groundwork for an abundance of analyses on this system, which will only further elaborate the capabilities of the virus.

*A. anophagefferens* presents a useful model given its designation as a harmful algal bloom (Figure 6B) and its status as one of the only organisms in its family with an isolated virus. Namely, bloom dynamics (especially the initiation process and termination factors—including the presence of the virus itself) present a useful and meaningful point of study *in situ*. This area is made all the more accessible by readily available strains of *A. anophagefferens*—both virus resistant and susceptible—with publicly accessible genomes. The host’s physiology has been intensely studied (e.g., nutrients and light effects on the host, effect of the host on other organisms in the environment), however, some interesting phenomena have yet to the fully characterized. This includes the role of the glycocalyx in infection, the algae’s transition to and metabolic capabilities in dormancy (Figure 6C), and its status as a mixotroph [i.e., evidence of heterotrophy (Figure 6D)]. How these relate to bloom formation and dynamics, overwintering, and interaction with virions remains to be elucidated. These algae-specific components are also not decoupled from the host-virus system, given that viral particles must also overwinter and no doubt the presence of dormant cells and the algae’s mixotrophy play a role in the host’s relationship to the virus. For example, can dormant cells be infected? Does a heterotrophic state cell permit viral lysis? A wealth of questions related to this system are emergent given the current foundational knowledge of both the virus and host.

Finally, attention must be paid to any algae implicated in harmful algal blooms. Not only is this a current issue, but also one that will only increase in prevalence given the changing climate. *A. anophagefferens* blooms are not an isolated phenomenon and have appeared in several independent areas geographically, with evidence of the ubiquitous nature of *A. anophagefferens* in general. Although brown tide bloom formation is a complex phenomenon, and no model predicts their appearance at present, background populations of *A. anophagefferens* cannot be exclusively ruled out as potential bloom seeds in new areas globally. Given the diverse nature of harmful algal blooms and their potential regulators, we recommend similar analyses as the ones described here be conducted on other lineages of algae infecting *Nucleocytoviricota*. The introduction of more systems for study in this diverse and variable phylum will only lead to a more complete understanding of bloom-virus dynamics. A continued understanding of this algae, and this host-virus system, will surely be valuable for our future approach to harmful algal blooms.

## Author contributions

AT: Conceptualization, Formal analysis, Writing – original draft, Writing – review and editing. EC: Conceptualization, Methodology, Writing – original draft, Writing – review and editing. EG: Data curation, Writing – review and editing. MM: Data curation, Writing – review and editing. BC: Data curation, Writing – review and editing. FA: Data curation, Writing – review and editing. CX: Data curation, Writing – review and editing. CG: Data curation, Writing – review and editing. SW: Conceptualization, Data curation, Funding acquisition, Project administration, Writing – review and editing.
